# Strategies for Increasing the Throughput of Genetic Screening: Lessons Learned from the COVID-19 Pandemic within a University Community

**DOI:** 10.3390/biotech13030026

**Published:** 2024-07-11

**Authors:** Fernanda Miguel, A. Raquel Baleizão, A. Gabriela Gomes, Helena Caria, Fátima N. Serralha, Marta C. Justino

**Affiliations:** 1 IPS COVID Lab, Instituto Politécnico de Setúbal, Rua Américo da Silva Marinho, 2839-001 Lavradio, Portugalgabriela.gomes@estbarreiro.ips.pt (A.G.G.); helena.caria@ess.ips.pt (H.C.); 2 RESILIENCE—Center for Regional Resilience and Sustainability, Escola Superior de Tecnologia do Barreiro, Instituto Politécnico de Setúbal, Rua Américo da Silva Marinho, 2839-001 Lavradio, Portugal; maria.serralha@estbarreiro.ips.pt; 3 MARE—Marine and Environmental Sciences Centre, Escola Superior de Tecnologia do Barreiro, Instituto Politécnico de Setúbal, Campus do IPS, Estefanilha, 2910-761 Setúbal, Portugal; 4 Departamento de Engenharia Química e Biológica, Escola Superior de Tecnologia do Barreiro, Instituto Politécnico de Setúbal, Rua Américo da Silva Marinho, 2839-001 Lavradio, Portugal; 5 BioISI—Instituto de Biosistemas e Ciências Integrativas, Faculdade de Ciências, Universidade de Lisboa, 1749-016 Lisboa, Portugal; 6 Departamento de Ciências Biomédicas, Escola Superior de Saúde, Instituto Politécnico de Setúbal, Campus do IPS, Estefanilha, 2914-503 Setúbal, Portugal

**Keywords:** COVID-19 pandemic, real-time reverse transcription quantitative PCR, sample-pooling, multiplex

## Abstract

Amidst the COVID-19 pandemic, the Polytechnic University of Setúbal (IPS) used its expertise in molecular genetics to establish a COVID-19 laboratory, addressing the demand for community-wide testing. Following standard protocols, the IPS COVID Lab received national accreditation in October 2020 and was registered in February 2021. With the emergence of new SARS-CoV-2 variants and safety concerns for students and staff, the lab was further challenged to develop rapid and sensitive diagnostic technologies. Methodologies such as sample-pooling extraction and multiplex protocols were developed to enhance testing efficiency without compromising accuracy. Through Real-Time Reverse Transcription Polymerase Chain Reaction (RT-qPCR) analysis, the effectiveness of sample pooling was validated, proving to be a clear success in COVID-19 screening. Regarding multiplex analysis, the IPS COVID Lab developed an in-house protocol, achieving a sensitivity comparable to that of standard methods while reducing operational time and reagent consumption. This approach, requiring only two wells of a PCR plate (instead of three for samples), presents a more efficient alternative for future testing scenarios, increasing its throughput and testing capacity while upholding accuracy standards. The lessons learned during the SARS-CoV-2 pandemic provide added value for future pandemic situations.

## 1. Introduction

The emergence of the novel coronavirus, named severe acute respiratory syndrome coronavirus 2 (SARS-CoV-2), precipitated the worldwide COVID-19 pandemic lasting from 2020 to 2022 [1]. By February 2021, there were over 105.4 million confirmed cases and 2.3 million fatalities. Those conducting diagnostic testing encountered numerous hurdles, primarily restricted to symptomatic cases. However, the substantial threat of transmission from asymptomatic carriers underscored the urgency of establishing extensive and affordable testing protocols. The higher education institutions (HEIs) were challenged to continue their educational programs, namely, professional or curricular training, to avoid disrupting students’ curricular activities. After the lockdown, with the possibility of engaging in normal curricular training at companies and health institutions, HEIs were faced with the responsibility of ensuring conditions for the students and academic staff’s safety and rapidly testing them to satisfy the security criteria of National Health Institutions.

Real-time reverse transcriptase quantitative polymerase chain reaction (RT-qPCR) analysis served as the primary diagnostic method due to its high sensitivity, capacity to detect viral infections even before the onset of symptoms, and ability to provide a 72 h safety window when yielding negative results [2]. However, the widespread use of RT-qPCR for COVID-19 diagnosis was hindered by challenges such as the extraction of viral RNA from oro/nasopharyngeal swabs and the time-consuming nature of RT-qPCR analysis, limited to approximately 30 samples per run (in a 96-well plate system), alongside reagent shortages, particularly in low-income regions [3]. Although rapid antigen tests and other fast detection methods were developed [4] and became available several months later, their initial sensitivity and reliability for early case screening were low, with results dependent on testing conditions [5]. Consequently, at the Polytechnic University of Setúbal (IPS), according to national policies [2], positive rapid antigen test results required further validation through RT-qPCR. Moreover, the IPS COVID Lab was instructed to report all positive results within 24 h of sample collection.

To overcome these obstacles, the IPS COVID Lab tested pooled samples as a promising solution for managing resources and simultaneously increasing the rapidity of test results and testing capacity. Initially developed during World War II for syphilis screening [6], pooled testing has since been used in the screening of infectious diseases, including HIV, influenza, malaria, and bacterial infections [3]. Pooling tests involve amalgamating samples into pools for analysis, followed by individual testing of positive pools (pool deconvolution). A negative pool result indicates that all samples within it are negative, enabling an increase in testing capacity without a corresponding increase in the number of tests conducted [7]. Successful implementation of pooled testing relies on essential factors such as population prevalence, test sensitivity and specificity, and the limit of detection. It is crucial to determine an optimal pool size that balances resource conservation with testing accuracy [8]. There are several pooling testing methods, including adaptive testing, Dorfman pooling, binary splitting, multistage testing, and household grouping [3]. The selection of an appropriate pooling strategy hinges on several factors, such as the specific objectives of the testing, available resources, and the demographics of the population under examination, with each approach having its advantages and drawbacks and the most appropriate choice depending on the context [3]. In the present paper, the Dorfman pooling strategy (a non-adaptive pooling approach) was selected. This technique involves combining samples from different individuals to form a composite sample, typically used in diagnostic testing to save consumables [3]. While this approach may sacrifice individual-level data when pooling occurs during sample collection, it proves particularly effective when disease prevalence is low.

Additionally, the IPS COVID Lab also developed Multiplex RT-PCR protocol methods to attain diagnoses more rapidly. This methodology consists of the simultaneous amplification of multiple products in the same PCR reaction using several primers and probes, reducing the total number of tubes/wells in the PCR analysis for each sample. In this situation, each probe should emit a specific fluorescence signal using different fluorophores to differentiate the targets [9,10,11]. Table 1 illustrates the different fluorophores and quenchers, with their corresponding excitation and emission wavelength ranges, used in the present work in real-time PCR techniques enabling their detection through different channels of the apparatus [12]. This approach has the advantage of reducing the number of individual reactions conducted for each subject and has been applied to several viruses, including SARS-CoV-2 (using other viral targets), avoiding multiple reactions for each individual test [9,10,11,13]. On the downside, this technique requires specific equipment capable of simultaneously detecting multiple different detection channels (different wavelengths), so that by reading at the wavelength of each fluorophore, different results can be obtained for the same reaction.

The present work describes a three-sample pooling strategy, the implementation of a multiplex protocol, and their validation to maximize COVID-19 screening within the Setubal Polytechnic University community. The main purpose was to conduct screening accurately and on time, enabling the identification of early cases to efficiently circumscribe potential outbreaks, ensuring the safety of students in their professional and curricular training and classes while effectively managing consumables and reducing plastic waste in the lab. Although originally tailored for the COVID-19 pandemic, this paper’s value can transcend its immediate context, and it can be applied to other emergent viral epidemical situations, such as the monkeypox virus, flu A, and avian flu, among others. The suggested screening strategies emphasize technology and community participation and demonstrate adaptability to diverse pandemic scenarios, albeit requiring some adjustments.

## 2. Materials and Methods

### 2.1. IPS COVID Lab Accreditation and Admission Proceedings

The IPS COVID Lab was registered by Portuguese Entidade Reguladora da Saúde (ERS) as a national laboratory for clinical analysis and diagnosis of COVID-19 in February 2021 after receiving national accreditation by Instituto Nacional de Saúde Doutor Ricardo Jorge (INSA) for its SARS-COV-2 analysis protocol in October 2020. All individuals that were tested at IPS COVID Lab completed a survey for informed consent and to schedule their sampling. This survey was validated by Polytechnic University of Setúbal, complied with the national policies for General Data Protection Regulation, and included a clause for sample storage, sharing, and use for research and investigation purposes.

### 2.2. Sample Collection and Storage

The initial recommendations of the World Health Organization [14] for sample collection were used, namely, performing both oropharyngeal and nasopharyngeal (both nostrils) swabs. Also, adopting a concentrating strategy [14], both sample swabs were placed in one tube containing 1 mL of viral inactivating transport medium (ITM—sample preservation fluid, Boer Technology, Frilabo, Lisboa, Portugal). This allowed the safe transport (<6 h) of the samples at room temperature. All tubes were externally disinfected with 15% bleach (*v*/*v*) to ensure safety in manipulation. Samples were left for at least 30 min at room temperature for viral inactivation and further heat-inactivated at 56 °C for 15 min prior to being analyzed.

A total of 4935 swab samples were collected from individuals and analyzed via RT-qPCR in this study. Within the time window of fall/winter 2021–2022, 4040 swab samples were tested, with sample pooling being applied to 3393 of these, resulting in a total of 1131 pooled RNA samples.

### 2.3. Sample Pooling and Procedure Validation

A procedure was developed to analyze 3 different samples simultaneously (pool). RNA was extracted using NZY Viral RNA Isolation Kit (NZYTech, Lisbon, Portugal), omitting the step of sample dilution with the inactivating buffer NVL, with the same composition as the ITM collection medium used and previously validated. Each pool of 600 µL was prepared by adding 100 µL of each of the 3 samples placed in ITM media into a sterile 1.5 mL microtube plus 300 µL of molecular-biology-grade ethanol 99.8 % (*v*/*v*) (Sigma-Aldrich, Merck, Algés, Portugal). For individual samples, the regular protocol was to use 200 µL of the swab fluid (in ITM medium) plus 200 µL of ethanol, with 100 µL of each sample being the kit’s manufacturer recommendation, allowing the samples to be loaded with a single centrifugation step. After homogenization, the pool mixture was then loaded into a mini spin column and centrifuged at 8000× *g* rpm for 1 min. The rest of the protocol was performed in accordance with the manufacturer’s recommendations. Succinctly, the spin-column was washed with 200 μL of NV buffer and 600 μL of NVW buffer, followed by 300 μL of NVW buffer. Then, the membrane was dried via centrifugation for 2 min at 8000 rpm, and the pooled RNA samples were eluted by adding 60 μL of ultrapure RNAse-free molecular-biology-grade water (NZYTech, Lisbon, Portugal).

RT-qPCR analysis was performed using NZYSpeedy One-step RT-qPCR Probe Master Mix (NZYTech, Lisbon, Portugal). Three reactions were prepared for each RNA sample (pool or individual), using the primers and probes for the targets recommended by the American Center for Disease Control and Prevention (US-CDC): a positive control for RNA quality, targeting the human gene RNase P (RP), and two targets of SARS-CoV-2, the US CDC N1 and N2 targets, within the viral N gene. The primers and probes used were 2019-nCoV Probe & Primer for SARS-CoV-2, a CDC-Qualified kit (Biosearch Technologies, Frilabo, Lisboa, Portugal). Each reaction mixture contained 5 μL of RNA sample, 400 nM of the primer pairs (forward and reverse), and 100 nM of the FAM/BHQ1-labeled probe in a total of 20 μL. For N2 reactions, salmon sperm ssDNA was added at 0.1 ng/μL. The reactions were performed in a Bioron RealLine 96-5 Cycler for Real-Time PCR (Bioron Diagnostics GmbH, Frilabo, Lisboa, Portugal) in 96-well plates using the following program: [(10 min at 50 °C); (5 min at 95 °C); 45× (5 s at 95 °C; 30 s at 55 °C)]. Afterward, the FAM channel and amplification curves were analyzed with Bioron’s RealTime_PCR v7.9 software to determine the crossing point (Cp) values. For an analysis to be considered valid, the RP gene curve should amplify (Cp ≤ 35). A positive test for SARS-Cov-2 was defined as having a Cp value ≤ 35 for both N1 and N2 targets. Conversely, a negative sample would exhibit no fluorescence curve for both N1 and N2 targets with curves of Cp > 35.

The number of samples in each pool was chosen in consideration of the ability to satisfy the need to deconvolute positive samples rapidly and predict the loss of sensitivity due to sample dilution. Optimization to validate pooling included the comparison of the Cp values obtained from individual positive samples with the Cp values obtained from pooling containing 2 negative samples and the former positive sample. Samples with different ranges of viral loads were analyzed, including those with low loads (Cp ~35). In cases where the presence of SARS-CoV-2 was detected in an RNA pool, the RNA extraction of each individual sample would be performed, followed by RT-qPCR, to assure the result.

### 2.4. Multiplex RT-qPCR and Validation

In this approach, the RT-qPCR was adapted for a multiplex protocol, maintaining the genomic targets (viral N1 and N2 sequences), along with the RP human target, as quality control. Primers and probes’ sequences were the same as in the standard methodology, and the only modification was to include in their synthesis (STAB Vida, Lda, Caparica Portugal) fluorophores and their respective quenchers for the probes, as detailed in Table 2. Primers and probes were provided in a lyophilized state (STAB Vida, Lda) and resuspended in ultrapure molecular-biology-grade water (NZYTech, Lisbon, Portugal) at a concentration of 100 µM. The preparation of the probes was carried out under light protection.

Three master solutions of primers + probe, with a final volume of 40 µL each, were prepared for each target (RP, N1, and N2), combining primer pairs at 25 µM, 6,25 µM of the probe, and molecular biology-grade water. To evaluate the simultaneous detection of multiple targets using multiplex PCR, several experiments were conducted and compared with the standard method, with the kit manufacturer’s recommendations being followed for multiplex reactions. The final primer and probe concentrations in multiplex reactions were lowered to 250 nM and 62.5 nM, respectively, as recommended.

In the assays, the number of targets analyzed simultaneously were altered, encompassing all three targets and different combinations of two targets, and, for controlling the efficiency with different fluorophores, simplex assays were also performed; 7 combinations of primer and probe samples were prepared. Depending on the number of targets to be analyzed in each sample, 8 µL of each master solution of the chosen target was added. The remaining volume was then adjusted with molecular-biology-grade water to a final volume of 40 µL, ensuring that each primer and probe were present in concentrations of 5 µM and 1.25 µM, respectively.

Multiplex procedures were characterized via the determination of the detection limit and the efficiency of the reactions. This involved preparing 8 successive 1/5 dilutions of positive control samples with artificial viral RNA (EURM019, European Commission Joint Research Center) or RNA from swab samples collected from non-infected individuals, using ultrapure water as solvent. Additionally, series of successive 1/5 dilutions of the artificial viral RNA were generated using a pool of RNAs extracted from swab samples of non-infected individuals as the solvent to confirm the virus detection limit when in the presence of human RNA contaminant background of swab samples. The analyzed viral RNA concentration ranged from 20,000 to 0.256 copies per microliter (cp/µL) in water and from 20,000 to 6.4 cp/µL in RNA extracted from uninfected individuals.

Once prepared, the reaction mixtures were dispensed into the PCR plate (15 µL per well), followed by the addition of 5 µL of each of the RNA dilutions. Alternatively, 5 µL of the solution used in the standard method served as a positive control (either viral RNA or human target gene), while 5 µL of water was added for the negative controls.

## 3. Results and Discussion

During mass testing for the return to in-person classes in the fall/winter of 2021, it was crucial for higher-education institutions to ensure efficiency and rapidity to, in turn, ensure community safety, prevent class disruptions, and mitigate chains of contagion. One specific challenge was the need for health students’ mass testing before their placement in healthcare institutions or workplaces, often involving up to 100 sample analyses per day in the IPS COVID Lab.

To enhance RNA extraction productivity for RT-qPCR detection while minimizing expenditure on RNA extraction kits during this mass-testing period, the IPS COVID Lab developed two distinct strategies. These strategies aimed to streamline the analysis process and guarantee accurate and trustworthy results, thereby facilitating a safe return to in-person activities amidst the pandemic.

### 3.1. Sample Pooling

The selection of a relatively small pool size of three samples per pool sample strategy was based on previous studies on sample pooling for COVID-19 RT-qPCR analysis. These studies have consistently shown that smaller pool sizes tend to maintain higher sensitivity compared to larger pools [15,16]. Additionally, considerations were made regarding the practicalities of deconvoluting positive samples and the workload capacity for processing them. It was determined that a smaller pool size of three samples would facilitate the deconvolution of positive results and accelerate the re-run process in RT-qPCR analysis. This strategic choice was intended to balance sensitivity and efficiency, ensuring accurate and timely results while optimizing laboratory workflow during mass-testing efforts. In pooled testing, if a pooled sample tests negative, then all samples within that pool are deemed negative. On the other hand, in cases where the presence of SARS-CoV-2 was detected in a pool, it had to be deconvoluted into its three constituent samples, and RNA extraction and RT-qPCR analysis were repeated for the individual samples. This also meant that the sooner people were warned about their test results, the greater the efficacy of activating measures to prevent infection and its spread [17,18].

**Figure 1:**
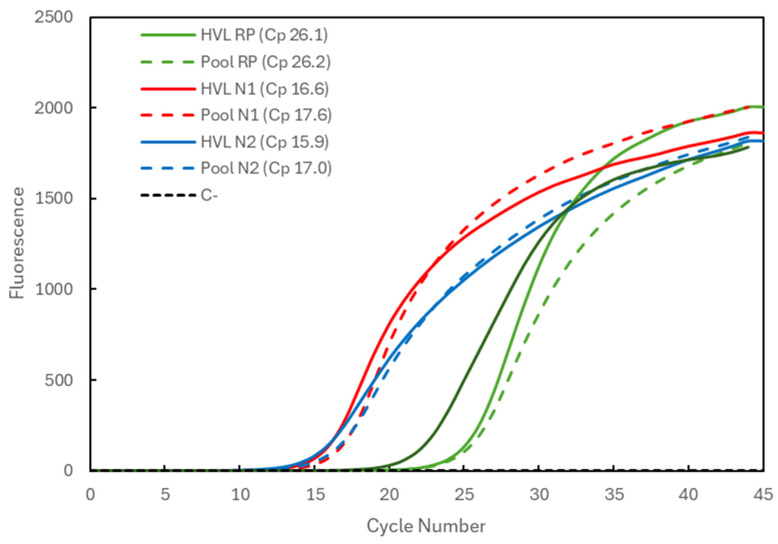
Comparison of Cp values for a high-viral-load (HVL) positive sample analyzed individually (solid lines correspond to HVL + target) and in a 3-sample pool (dashed lines correspond to Pool + target). The 3 genetic targets were the RP human gene (green lines) and viral N1 (red lines) and N2 (blue lines) targets. For each reaction, the Cp value is presented. The dashed black line (C^−^) represents the negative control reaction induced for each target, illustrative of all three.

In Figure 1, the Cp comparison is presented for a sample with high viral load (HVL). When analyzed individually (full lines), the Cp values were 26.1 for the RP gene (green) and 16.6 and 15.9 for the N1 and N2 targets (red and blue), respectively, of the SARS-CoV-2 virus. When pooled with two other negative samples, the Cp values (dashed lines) remained constant for the RP gene (26.2) and increased to 17.6 and 17.0 for the N1 and N2 targets, respectively. The average Cp value for the RP gene at IPS COVID Lab was determined to be 26.69 ± 1.03 (n = 4935).

**Figure 2:**
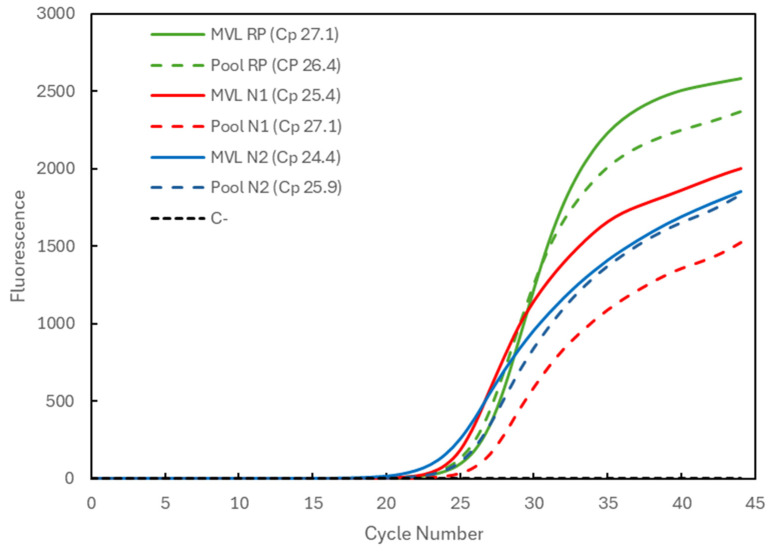
Comparison of Cp values for a medium-viral-load (MVL) positive sample analyzed individually (solid lines correspond to MVL + target) and in a 3-sample pool (dashed lines correspond to Pool + target). The 3 genetic targets were the RP human gene (green) and the viral N1 (blue) and N2 (red) targets. For each reaction, the Cp value is presented. The dashed black line (C^−^) represents the negative control reaction induced for each target, illustrative of all three.

Figure 2 compares the results for a positive sample with medium viral load (MVL) individually processed (solid lines) and for the three-sample pool (dashed lines) combined with two negative samples. The Cp values obtained for the individually analyzed sample were 27.1 for the RP gene (green), 25.4 for the N1 target (red), and 24.4 for the N2 target (blue). The Cp values for the pooling were 26.4 for the RP gene (within the average) and again slightly higher for the viral targets, amounting to 27.1 and 25.9 for the N1 and N2 targets.

**Figure 3:**
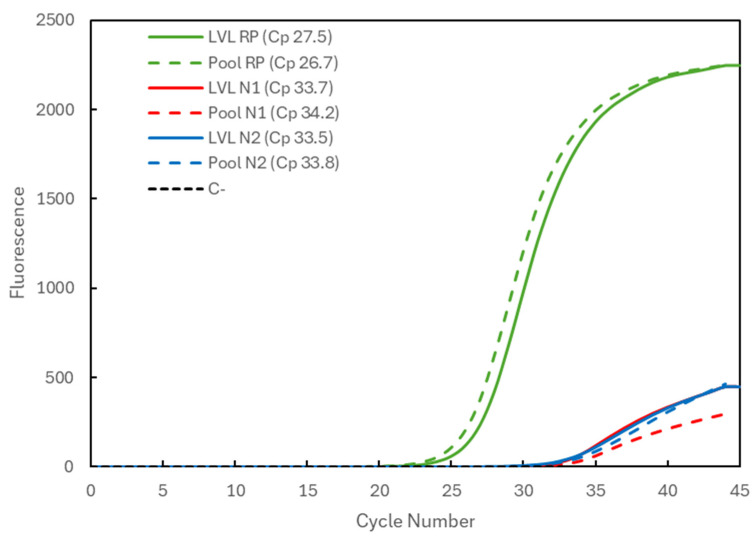
Comparison of the Cp values for a very-low-viral-load (LVL) positive sample analyzed individually (solid lines correspond to LVL + target) and in a 3-sample pool (dashed lines correspond to Pool + target). The 3 genetic targets were the RP human gene (green) and viral N1 (blue) and N2 (red) targets. For each reaction, the Cp value is presented. The dashed black line (C^−^) represents the negative control reaction induced for each target, illustrative of all three.

In Figure 3, the results are shown for a positive sample with a very low viral load (LVL). Corresponding to the analysis of the samples individually (solid lines indicate LVL), it presents the Cp values of 27.5 for the RP gene (green) and 33.7 and 33.5 for the N1 (red) and N2 (blue) targets of the viral N gene, respectively. When the sample was analyzed in a pool with two negative samples (dashed lines indicate Pool), the Cp values were 26.7 for the RP gene and 34.2 and 33.8 for the N1 and N2 targets, respectively. Again, an increase in the viral Cp values was seen, but it was within a window of approximately +1 in Cp value, i.e., a two-times lower concentration of the target, with RP gene values within the range.

Our results show that based on the RT-qPCR analysis of Cp data, a reduction in sample quantity from 200 µL (when processed individually) to 100 µL (in a pool) does not affect PCR sensitivity or the conclusions drawn. In general, the Cp values for the human RP gene are within the average, suggesting that the process of extraction was not affected by the increase in the amount of sample loaded into the column. There was a consistent slight delay in capturing the positive signal, resulting in an increase in Cp values for the N1 and N2 targets of the SARS-CoV-2 N gene. This increase (+1 Cp unit) is within the expected range considering the effect of the dilution effects (approx. ½), and it was observed for samples with both high and low viral loads, with lower Cp values indicating earlier amplification and higher Cp values indicating later amplification still considered positive.

The obtained results validate the use of pooling for COVID-19 screening tests using the RT-qPCR technique, reducing result response times, extraction kit expenses, and plastic waste production, constituting an optimization for mass testing that could be applied in future emergent situations.

The sensitivity of RT-qPCR is influenced by multiple factors, including kit sensitivity, dilution levels, sample collection techniques, sample types (nasopharyngeal, oropharyngeal, nasal, saliva, etc.), sample transport temperature, and the varying viral load at different stages of the disease [19,20].

In our work, we consistently used the same type of sample, and the sample collection technique (oropharyngeal plus nasopharyngeal swabbing of both nostrils, with the material collected into the same tube) was chosen specifically to enrich viral concentration. The collection medium chosen (ITM) was an RNA stabilizing solution that reduces the risks associated with sample transport temperature and viral RNA degradation, aspects particularly relevant as most samples were collected on an IPS campus 30 km away from the campus where the laboratory was situated. In over 1000 prior samples, RNA was extracted successfully, suggesting that our method ensures the swab samples’ RNA integrity. As for the protocol used and particularly the kit’s sensitivity, the IPS COVID Lab received national accreditation to perform COVID-19 diagnoses, within the lower limits established by INSA.

Several authors have highlighted that sample pooling strategies still suffer from inherent limitations as they cannot guarantee the diagnostic accuracy of individual samples and may hide technical errors such as insufficient sampling [18,21], which might increase the likelihood of acquiring false negatives, decreasing sensitivity and raising the risk of overlooking samples with weak viral loads or borderline positivity due to sample dilution [18,22,23,24]. Thus, while sample pooling optimizes resource utilization, it runs the risk of overlooking individuals who may test positive for COVID-19 [23,25].

Accordingly, we opted for a small pool size to mitigate the challenges mentioned, and while nationally accredited for diagnosis of low viral loads, our protocol and equipment analyzed beyond the 35-threshold cycle number to decrease the possibility of acquiring false negatives even further. Prior assays for the RT-qPCR analysis had shown a limit of detection of 6.4 viral RNA copies/μL (see Section 3.2). Moreover, in very diluted samples, the amplification of the viral targets could be detected with Cp values up to 37, i.e., 2 Cp units above the recommended threshold of 35 cycles for diagnostic purposes. So, even if a pooled sample exhibited amplification of viral targets with a Cp higher than 35 cycles, it would still be possible to identify it as a borderline positive and deconvolute it to confirm and identify the low viral load sample within the pool.

Striking a balance is crucial when weighing the benefits and costs associated with reduced accuracy, and countries and the public health sector must consider these factors when determining optimal testing strategies. This information is vital for determining pooled testing strategies, particularly for diseases like COVID-19. In the COVID-19 pandemic scenario, missing any positive cases was dangerous. As D’Arienzo and Coniglio state, given the highly contagious nature of SARS-CoV-2, every overlooked positive case could contribute to the rapid spread of infection and outbreaks [26].

Within the period of fall/winter 2021–2022, we employed this pooling strategy in approximately 84% of tests, and from the 1131 pools analyzed, 25 (2%) were deconvoluted. To minimize false negatives, the IPS COVID Lab used stringent criteria for deconvolution to include suspicious samples with borderline Cp values of the viral targets or one valid positive result on one of the viral targets. Of these 25 deconvoluted pools, only 16 (64%) proved to be positive for COVID-19.

These numbers seem strangely low, but a secondary strategy was used to avoid the necessity of deconvolution; i.e., an efficient triage and risk assessment was performed, and all subjects showing symptoms or being at high risk due to direct exposure (unmasked) to COVID-19-infected patients were not pooled.

### 3.2. Multiplex RT-qPCR

Developing a Multiplex RT-qPCR *in-house* protocol was the second strategy followed, keeping the viral targets N1 and N2, along with the human control gene RP, to increase the SARS-COV-2 analysis throughput, since each PCR plate only allows the analysis of about 30 samples in the standard procedure, with three wells per sample to individually test the RP, N1, and N2 targets.

Identical primers (to those used in the standard procedure) with distinct fluorophores (FAM for N1, HEX for RP, and ROX for N2), were used, and a comprehensive array of combinations featuring several genomic targets was devised, allowing the assessment of their performance both individually and in combination, including in duplex and triplex configurations. The triplex solution encapsulated all three genetic targets, while separate duplex solutions were formulated for pairs as N1 + N2, RP + N1, and RP + N2, alongside simplex solutions for each target.

**Figure 4:**
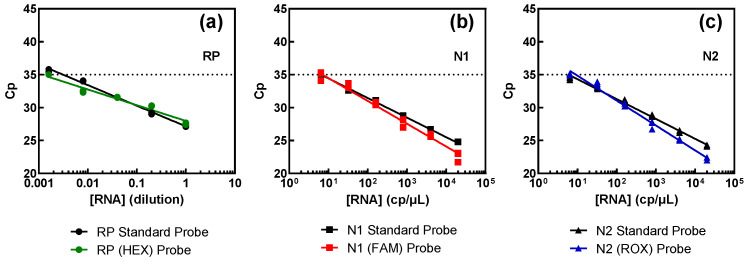
Assessment of the probes for the multiplex procedure versus the FAM-labelled probes of the standard procedure for each genomic target: RP (**a**), N1 (**b**), and N2 (**c**). Pure SARS-CoV-2 RNA (N1 and N2 targets) at 2000 copies per microliter (cp/μL) and swab-derived human RNA (RP target), with an estimated initial value of 1, were successively diluted in water.

Results obtained from the comparison between these samples and the standard procedure are illustrated in Figure 4, and juxtapose the outcomes obtained with the standard method (depicted in black) and samples containing the new probes (shown in color). The results show that all three targets exhibited comparable performance, irrespective of the fluorophores, indicating that different fluorophores do not influence detection capability.

The assertion mentioned above is corroborated by the efficiency results, consistently hovering around 100% across all targets, as summarized in Table 3. As can be seen, the probes prepared for multiplex detection show a similar behavior to the standard ones. For the RP target, the number of copies present is unknown as they were obtained from a pool of RNA extracted from several nasopharyngeal or oropharyngeal swabs. The limit of detection is lower than that of the viral targets but was the equivalent of a ~600-fold dilution of a regular swab sample.

Table 4 illustrates the results of multiplex reactions induced with combinations of the three targets—triplex and duplex RP + N1, RP + N2, and N1 + N2. The linear relationship between Cp and the concentration logarithm was somewhat lost, probably due to competitive effects that have been reported to lead to efficiencies significantly different from the ideal/standard procedure [27]. When the dilutions were made in a matrix of RNA extracted from swab samples, it was observed that all targets were 100% detected up to an RNA dilution of 160 copies of viral RNA per µL. For the samples prepared with dilutions in water, the RP target stops being detectable, as expected, from the dilution corresponding to 800 copies of viral RNA per µL, in the duplexes and in the triplex samples. Competition effects in the reactions, when the other targets are at higher concentration [27], may justify the detection limit being higher than in RP simplex reactions (Table 3 and Figure 4). For a viral RNA concentration of 32 cp/µL, the RP was fully detected in samples diluted in the RNA matrix, whether in triplex or duplex reactions, as it is the more abundant template, whereas viral amplification failed partially or totally for triplex and duplex samples, except for the N1 + N2 duplex combination when competition against human RNA template does not occur. For a viral RNA concentration of 6.4 cp/µL, viral detection was partial when samples were diluted in water for the N1 + N2 set and failed completely under all other conditions.

These results are in accordance with the expected competitive effects that the asymmetric sample composition has during the reactions, especially affecting the detection of the lowest-concentration targets, when targets have significantly different amounts [27]. Thus, under conditions where viral RNA is more abundant than human RNA, the simultaneous determination of RP is affected, potentially invalidating the results due to the lack of this control being positive. On the other hand, in situations where viral load is very low, the simultaneous amplification of the RP target affects virus detection, which might lead to false negatives, with dangerous effects for the human population [26]. We further analyzed a collection of positive samples diagnosed via the standard method. When the samples exhibited original Cp values of 15–20 for viral targets, both triplex and N1 + N2 duplex methods allowed 100% viral detection, but 8/11 samples failed to amplify the RP target with the triplex method. The same was seen for samples exhibiting original Cp values of 20–25, and in this case, only 1/8 samples failed to amplify the RP target with the triplex method. When the range of original Cp values was 25–30, the N1 + N2 duplex allowed 100% viral detection, whereas with the triplex method, viral detection was only possible in 10/13 samples. As for the very-low-viral-load positive samples, with original Cp values of 30–35, the triplex method failed totally, while with the duplex method, detection was possible in only 5/11 samples. Hence, the N1 + N2 duplex RT-qPCR method appears to be more reliable than the triplex method, but its sensitivity is much lower than the standard procedure used. The risk of false negatives led us to conclude that the multiplex approach developed in this work could be applied with an N1 + N2 duplex and the RP target separately for samples in which high viral loads are expected, e.g., for the required PCR confirmation of positive rapid antigen test results obtained during or in a short temporal window after sample collection. National health policies for COVID-19 diagnosis maintained for a long period of time the need to confirm via PCR testing all positive results of rapid antigen tests, not only the self-tests but also those performed by health professionals in registered establishments [2].

**Figure 5:**
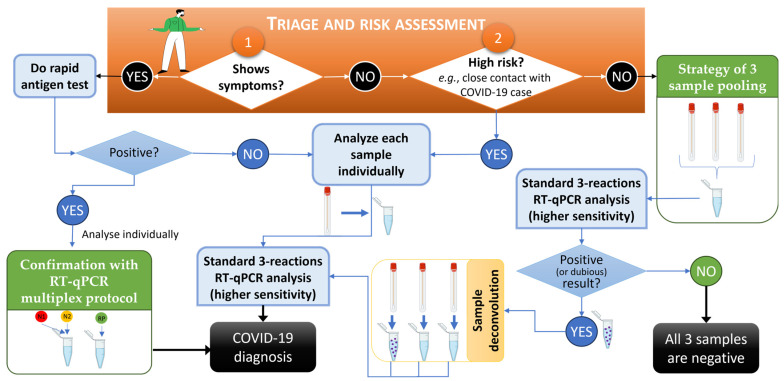
Workflow for COVID-19 mass screening using RT-qPCR-based diagnosis and relying on the developed strategies of low-risk sample pooling and multiplex analysis for predictably high-viral-load samples. In the orange box, numbers 1 and 2 represent the start of the procedure of triage and risk assessment of individuals. The pathways for Yes and No indicate the approach to be followed. Developed methodologies described in this work are in green boxes, and standard procedures are in light blue boxes. An RT-qPCR was considered dubious when at least one viral target was amplified and/or had Cp values above the standardized 35-cycle threshold. This workflow was created using BioRender.com and PowerPoint.

The presented strategies for increasing the throughput of genetic screening highlight the advantage of rapid screening strategies for the effective mitigation of COVID-19 or others viral epidemics and are in accordance with work by other authors who applied similar approaches to, e.g., monkeypox [28,29]. A workflow of the developed strategies of low-risk sample pooling and multiplex analysis for predictably high-viral-load samples for COVID-19 mass screening using RT-qPCR-based diagnosis is provided in Figure 5. In this workflow, it was found that an efficient triage and risk assessment of individuals prior to sample collection, carried out to choose sample processing sequencing, was the key starting point (orange box in Figure 5), with numbers 1 and 2 representing the starting point options according to patient symptoms and context. The developed methodologies described in this work are in green boxes, while standard procedures are in light blue boxes.

In summary, when individuals were symptomatic, besides the oro/nasopharyngeal swabs collected for PCR analysis, a rapid antigen test was immediately performed, thus allowing fast notification and activation of containment strategies. Confirmation via RT-qPCR was mandatory as per national policies [2], but in such cases, the multiplex procedure, even if less sensitive, could be used without concerns given its detection limit. Low-risk samples were analyzed with the pooling strategy, but the interpretation of RT-qPCR results followed more stringent criteria to minimize the risk of false negatives; i.e., besides all positive results, sample deconvolution and re-testing was performed when at least one viral target was amplified and/or had Cp values above the 35-cycle threshold (considered “dubious”). As mentioned earlier, of all the deconvoluted and re-tested samples, only 64% contained a positive sample. This workflow, in combination with both approaches, summarizes the advantage and relevance of the method presented in this study.

## 4. Conclusions

The onset of the COVID-19 pandemic in late 2019, driven by the novel coronavirus SARS-CoV-2 [1], swiftly swept across the globe, culminating in 34,627 confirmed cases and a 2% fatality rate by March 2020, prompting the implementation of widespread lockdown measures. As in-person activities resumed at IPS in the fall of 2021, community screening became imperative to curb potential infection chains while ensuring the safety of all academic activities. To meet this challenge, the IPS COVID Lab optimized COVID-19 diagnostic procedures and the workflow illustrated in Figure 5. The three-sample pooling method, alongside the multiplex of duplex protocol (N1 + N2 and RP), were successfully developed as strategies for conserving time and resources, with limited applications (concerning the screening of the low-risk population and the confirmation of positive antigen test results, respectively). The results obtained validate their efficacy in detecting the virus across varying viral loads. These methodologies were deployed during the fall/winter of 2021–2022, enabling the screening of approximately 4000 individuals and identifying 143 positive cases (3.5%), 25 of which exhibited very low or borderline-positive results. Notably, over 85% of the COVID-19 tests performed by the IPS COVID Lab during this period used RT-qPCR tests instead of rapid antigen tests, and, of these, 84% were analyzed with sample pooling. With an efficient triage and risk assessment (as illustrated in Figure 5), only 2% of the pooled samples required deconvolution and re-testing. To minimize the risk of false negatives, a stringent policy for classifying suspected positive pooled samples was employed, so only 64% of the deconvoluted pooled samples proved to contain at least one positive case, while the others were confirmed individually as being negative. The swift detection, targeted class/service screening, and containment of cases contributed to the absence of official outbreaks within the Polytechnic University of Setúbal *campi*.

While concerning the COVID-19 pandemic, this work can lay the foundation for adapting efficient mass-testing solutions to other situations of viral epidemics with a worldwide spread in the near future, wherein rapid positive case identification would be a relevant public health measure.

## Figures and Tables

**Table 1:** Fluorophores used with respective excitation and emission wavelengths (nm) and quenchers [12].

Fluorophore	Excitation Max (nm)	Emission Max (nm)	Extinction Coefficient *	Color **	Quencher
FAM	495	520	75,850	Yellow-Green	BHQ-1
HEX	535	556	98,000	Yellow	TAMRA
ROX	575	602	82,000	Orange-Red	BHQ-2

* Extinction coefficient at λ(max) in cm^−1^M^−1^. ** Typical emission seen through the eyepiece of a conventional fluorescence microscope with appropriate filters.

**Table 2:** Representation of the probes used for each genomic target in the multiplex technique.

Gene Target	Fluorophore 5′	Sequence (5′ → 3′)	Quencher 3′
RP	HEX	TTC TGA CCT GAA GGC TCT GCG CG	TAMRA
N1	FAM	ACC CCG CAT TAC GTT TGG TGG ACC	BHQ-1
N2	ROX	ACA ATT TGC CCC CAG CGC TTC AG	BHQ-2

**Table 3:** Efficiency of amplification of each target using standard vs. multiplex probes.

	RP ^1^		N1		N2	
Probe	Standard	Multiplex	Standard	Simplex	Standard	Simplex
Efficiency	109 ± 2%	126 ± 14%	117 ± 3%	95 ± 4%	114 ± 3%	84 ± 2%
Detection limit (Cp < 35)	DF ^2^ 625	DF ^2^ 625	≈6.4 cp/µL ^3^ DF ^2^ 15,625	≈6.4 cp/µL ^3^ DF ^2^ 15,625	≈6.4 cp/µL ^3^ DF ^2^ 15,625	≈6.4 cp/µL ^3^ DF ^2^ 15,625

^1^ The template for target RP was a pool of RNA samples extracted from human oro/nasopharyngeal swab samples, and the number of copies is unknown. An arbitrary value of 1× concentration is attributed to the non-diluted sample. ^2^ DF is dilution factor. ^3^ cp/µL stands for copies of SARS-CoV-2 RNA per µL.

**Table 4:** Results for the triplex and duplex assays.

[RNA] (cp/µL)	Dilutions Performed in RNA Matrix *				Dilutions Performed in Water			
	Triplex (RP + N1 + N2)	Duplex (N1 + N2)	Duplex (RP + N1)	Duplex (RP + N2)	Triplex (RP + N1 + N2)	Duplex (N1 + N2)	Duplex (RP + N1)	Duplex (RP + N2)
20,000	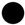	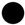	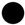	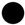	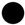	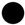	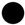	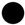
4000	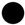	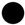	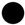	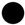	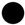	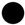	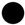	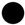
800	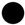	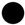	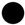	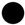	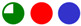	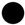	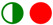	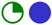
160	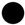	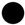	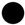	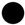	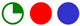	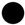	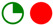	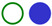
32	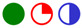	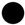	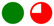	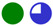	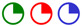	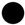	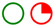	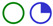
6.4	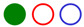	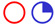	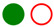	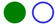	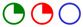	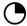	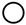	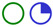

* From swab samples of non-infected individuals. Colors: black—all targets present in the assay; green—RP target; red—N1 viral target; blue—N2 viral target. 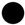
—100% of the target is present; 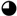
—75% of the target is present; 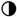
—50% of the target is present; 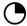
—25% of the target is present; and 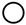 0% is present (no target detected) from a total of 4–6 replicates.

## Data Availability

The original contributions presented in the study are included in the article; further inquiries can be directed to the corresponding author.
